# A conserved RAD6-MDM2 ubiquitin ligase machinery targets histone chaperone ASF1A in tumorigenesis

**DOI:** 10.18632/oncotarget.5011

**Published:** 2015-08-22

**Authors:** Chen Wang, Jian-Feng Chang, Hongli Yan, Da-Liang Wang, Yan Liu, Yuanya Jing, Meng Zhang, Yu-Long Men, Dongdong Lu, Xiao-Mei Yang, Su Chen, Fang-Lin Sun

**Affiliations:** ^1^ Research Center for Translational Medicine at East Hospital, Tongji University, Shanghai, 200120/200092, China; ^2^ School of Life Sciences and Technology, Tongji University, Shanghai, 200120/200092, China; ^3^ UN School of Environmental Sciences and Technology, Tongji University, Shanghai, 200120/200092, China; ^4^ Department of Laboratory Medicine, Changhai Hospital, The Second Military Medical University, Shanghai, 200433, China; ^5^ Institute of Epigenetics and Cancer Research, School of Medicine, Tsinghua University, Beijing, 100084, China; ^6^ Department of Science and Education, People's Hospital of Zunhua, Tangshan, Hebei, 064200, China

**Keywords:** RAD6, MDM2, ASF1A, protein degradation, tumorigenesis

## Abstract

Chromatin is a highly organized and dynamic structure in eukaryotic cells. The change of chromatin structure is essential in many cellular processes, such as gene transcription, DNA damage repair and others. Anti-silencing function 1 (ASF1) is a histone chaperone that participates in chromatin higher-order organization and is required for appropriate chromatin assembly. In this study, we identified the E2 ubiquitin-conjugating enzyme RAD6 as an evolutionary conserved interacting protein of ASF1 in *D. melanogaster* and *H. sapiens* that promotes the turnover of ASF1A by cooperating with a well-known E3 ligase, MDM2, via ubiquitin-proteasome pathway in *H. sapiens*. Further functional analyses indicated that the interplay between RAD6 and ASF1A associates with tumorigenesis. Together, these data suggest that the RAD6-MDM2 ubiquitin ligase machinery is critical for the degradation of chromatin-related proteins.

## INTRODUCTION

The accurate organization of chromatin from DNA and histones is essential for almost all types of life processes, such as cell proliferation, differentiation and migration. The nucleosome is the repeat unit of chromatin and contains approximately 147 bp DNA and the core histones (H3, H4, H2A and H2B) [1–4]. These core histones form an octamer around which the DNA is wrapped. The nucleosome is a highly dynamic structure. The removal (disassembly) and deposition (assembly) of histones frequently occur during replication, gene transcription, DNA damage repair and recombination. Chromatin assembly is tightly regulated and mediated by histone chaperones, which function through binding to histones [5–8].

Anti-silencing function 1 (ASF1) is an H3/H4 chaperone that regulates chromatin assembly in both replication-dependent and replication-independent manners by interacting with chromatin assembly factor 1 (CAF-1) and histone regulatory homolog A (HIRA) proteins, respectively [9–13]. During DNA replication in human cells, ASF1A and ASF1B bind to the MCM2–7 complex, the helicase that unwinds DNA ahead of the replication fork, and evict H3/H4 histones from the front of the replication forks and transfer them to CAF-1. CAF-1 further deposits H3/H4 onto newly synthesized DNA strands [14]. The eviction of old histones by ASF1 also facilitates transcription factor or RNA polymerase II entry to the transcription start site at various promoter regions [15]. In addition, ASF1 mediates chromatin disassembly on gene promoter regions during transcriptional activation and elongation in budding yeast [16]. It has been reported that ASF1 binds to histones not bound to DNA, indicating its role in chromatin higher-order organization [17–19].

ASF1 is a highly conserved protein in eukaryotes and was first identified by its ability to overcome transcriptional silencing when overexpressed in yeast [20]. ASF1 has two isoforms in mammalian cells, ASF1A and ASF1B. The N-terminus of these two proteins is highly conserved and essential for the binding of H3.1-H4 replicative histones or H3.3-H4 replacement histones [21–23]. However, the biological functions of these two isoforms are different. For example, ASF1B is the ASF1 isoform necessary for cell proliferation in breast cancer, while ASF1A is not required in this process [24]. ASF1A, not ASF1B, determines H3K56Ac levels in human cells and functions in tumorigenesis [25].

Increasing evidence indicates that ASF1 plays important roles in many biological processes, such as the regulation of tumor formation and malignancy and DNA damage repair [8, 11, 13, 24, 25]. For instance, H3K56Ac, a downstream target of ASF1, is incorporated into DNA repair foci by ASF1 and histone acetyltransferase [25–28]. ASF1 binds to newly synthesized H3/H4 dimers and presents them for acetylation by Rtt109 in yeast [26, 28, 29]. The acetylation of histone H3 at lysine 56 promotes histone deposition by CAF-1 and Rtt106 in yeast as a result of a higher binding affinity of acetylated H3 to CAF-1 and Rtt106 compared with non-acetylated H3 [30–32]. H3K56Ac is also believed to be a critical histone marker for the maintenance of genomic stability [33] and also involved in transcriptional regulation [34].

Above all, ASF1 is a critical factor that functions in many biological processes. However, the regulation of ASF1 turnover remains unclear, especially in mammals [35]. In this study, we found for the first time that the RAD6-MDM2 ubiquitin ligase machinery regulates ASF1A degradation via the ubiquitin-proteasome pathway. RAD6 is an E2 ubiquitin-conjugating enzyme involved in H2B monoubiquitination [36–38], and MDM2 is a well-known E3 ligase for the degradation of p53 and other targets [39–44]. In addition to our previous work showed that the RAD6-MDM2 complex synergistically targets p53 for turnover [45–49], we found that ASF1A is a new and conserved target of the RAD6-MDM2 degradation machine in both *Drosophila* and *Homo sapiens*. We also found that this regulation participates in the process of tumorigenesis.

## RESULTS

### RAD6 interacts with ASF1A and regulates its degradation in Homo sapiens

**Figure 1:**
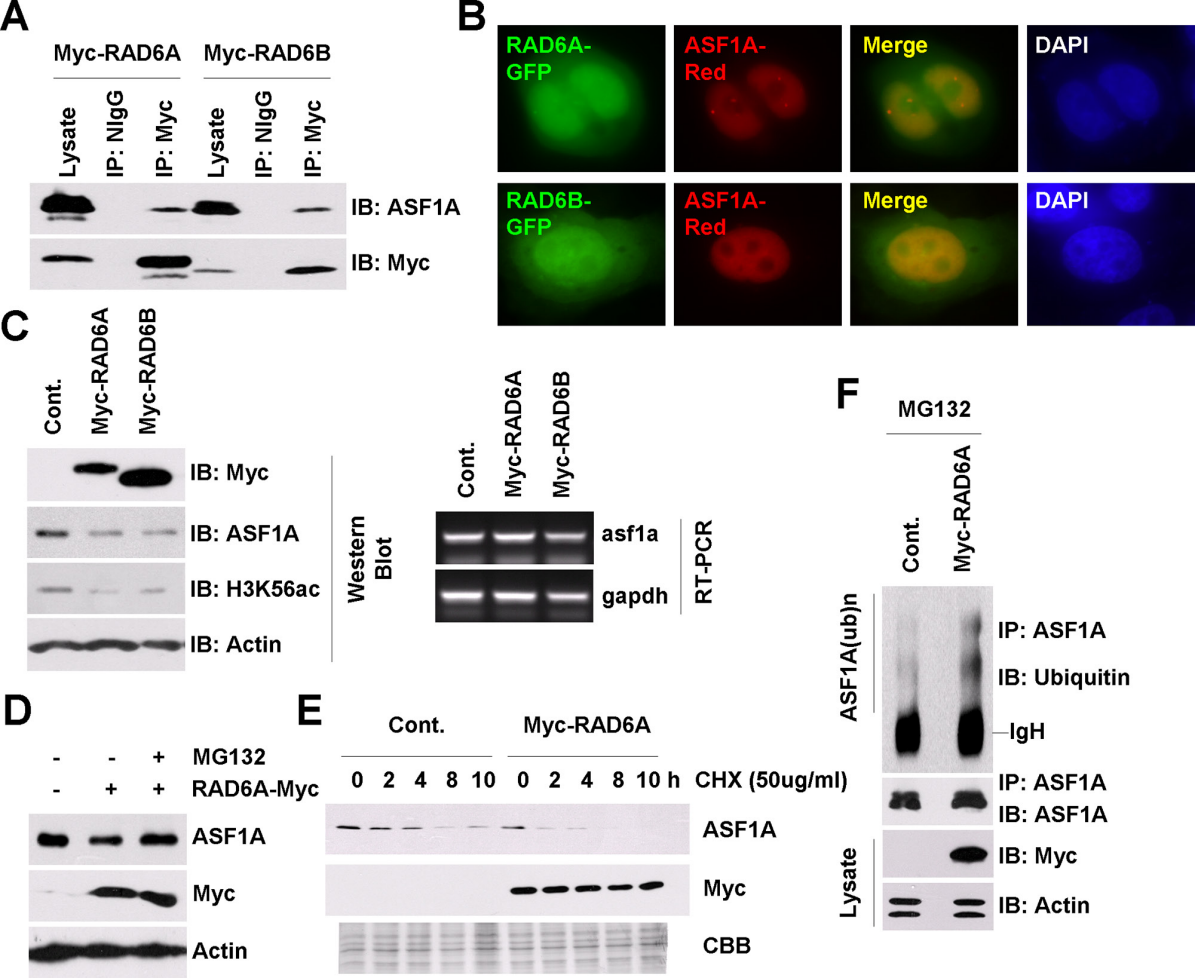
RAD6 regulates ASF1A ubiquitination and degradation in *Homo sapiens* **A.** RAD6 interacts with ASF1A *in vivo*. HL-7702 cells were transfected with Myc-tagged RAD6A and RAD6B for 48 h. Cell extracts were then prepared and subjected to a co-immunoprecipitation assay with antibodies against anti-Myc or anti-NIgG (normal IgG antibody, as a negative control). Western blot assays were performed with an anti-ASF1A antibody to detect the interaction between RAD6 and ASF1A. **B.** RAD6 colocalizes with ASF1A in HL-7702 cells. HL-7702 cells transfected with RAD6A-GFP or RAD6B-GFP together with ASF1A-Red were stained with DAPI. **C.** RAD6 regulates ASF1A protein levels and H3K56Ac levels. HL-7702 cells were transfected with or without Myc-tagged RAD6A or RAD6B for 48 h. The cells were lysed for western blot assays with antibodies as indicated (upper). HL-7702 cells were transfected with or without Myc-tagged RAD6A or RAD6B for 48 h. Total RNA was isolated and subjected to an RT-PCR assay with primers specific for *asf1a* and *gapdh* genes (lower). **D.** The regulation of ASF1A protein levels by RAD6 is mainly through the 26s proteasome pathway. HL-7702 cells transfected with or without Myc-tagged RAD6A for 48 h were treated with or without 25 μM MG132 for another 10 h. Cell extracts were then prepared and subjected to western blot assays with antibodies as indicated. **E.** RAD6 promotes ASF1A degradation. HL-7702 cells were transfected with or without Myc-tagged RAD6 for 48 h. The cells were then incubated with 50 μg/mL CHX for the indicated times. Cell lysates were prepared and subjected to western blot assays with antibodies as indicated. **F.** RAD6 promotes ASF1A ubiquitination. HL-7702 cells were transfected with or without Myc-tagged RAD6A for 48 h. The cells were then incubated with 25 μM MG132 for another 10 h. An immunoprecipitation experiment was performed using an anti-ASF1A antibody under denaturing conditions. An anti-ubiquitin antibody was used in a western blot assay to detect the ubiquitinated form of ASF1A.

It has been previously reported that ASF1 and Rad6 have a genetic interaction in yeast, especially after DNA damage [50]. We also found that dASF1 is a potential interaction partner of dRad6 from our yeast two-hybrid screening experiments using dRad6 as bait (data not shown). We therefore determined whether RAD6 interacts with ASF1 in *Homo sapiens*. We first employed a co-immunoprecipitation assay to investigate the binding between RAD6 and ASF1. HL-7702 cells were transfected with Myc-tagged RAD6A or Myc-tagged RAD6B for 48 h. Cell extracts were prepared and subjected to a Co-IP assay with an anti-Myc antibody; then, the precipitated proteins were subjected to western blot assays with an anti-ASF1A or anti-Myc antibody, as indicated. From the results shown in Figure 1A, we found that both RAD6 isoforms, RAD6A and RAD6B, interacted with ASF1A *in vivo*. Moreover, we also examined the subcellular distributions of both RAD6 and ASF1A. HL-7702 cells were transfected with the RAD6A-GFP or RAD6B-GFP plasmid together with the ASF1A-Red plasmid as indicated for 48 h. The cells were then harvested and stained with DAPI. Our results showed that both RAD6A and RAD6B colocalized with ASF1A in HL-7702 cells (Figure 1B). Our quantification suggested that almost all the transfected cells show this kind of colocalization pattern (data not shown). In addition, we also examined whether RAD6 interacts with other histone chaperons by using histone chaperon NASP as a subject. From our Co-IP assay, our results showed that RAD6 did not interact with NASP in human HEK293T cells, but the interaction between RAD6 and ASF1A can also be detected in HEK293T cells (Supplementary Figure S1), suggesting the specificity of the interaction between RAD6 and ASF1A in human cells.

We and other groups previously showed that RAD6 participates in the degradation process of several important proteins [45–49]. To address the function of this interaction, we first examined the effect of RAD6 on ASF1A protein levels. As shown in Figure 1C, RAD6 overexpression indeed downregulated ASF1A protein levels significantly (Figure 1C, western blot, upper), while there was no obvious change in ASF1A mRNA levels (Figure 1C, RT-PCR, lower). It has been reported that ASF1A is a key regulator of the acetylated histone H3 at lysine 56 (H3K56Ac) [25–28]. We also tested whether RAD6 further affects the H3K56Ac levels. Indeed, as a downstream epigenetic marker of ASF1A, H3K56Ac levels were decreased strikingly in RAD6 overexpressing cells (Figure 1C, western blot, upper). This result indicated that RAD6 downregulates ASF1A protein levels, likely via a posttranscriptional pathway.

To identify whether the control of ASF1A protein levels by RAD6 is through the ubiquitin-proteasome pathway, we employed MG132 to inhibit the activity of the 26s proteasome. HL-7702 cells transfected with an empty vector (−) or with a Myc-tagged RAD6A (+) for 48 h were treated with DMSO (−) or with 25 μM MG132 (+) for 10 h. The cells were then lysed for western blot assays. We found that MG132 treatment inhibited the RAD6 overexpression-induced ASF1A decrease (Figure 1D). We further performed a chase assay to determine the effect of RAD6 on the ASF1A protein half-life. As shown in Figure 1E, RAD6 overexpression decreased the ASF1A half-life time significantly, indicating an accelerated ASF1A degradation.

Finally, we employed an *in vivo* ubiquitination assay to test the effect of RAD6 on ASF1A ubiquitination. HL-7702 cells were transfected with an empty vector (Cont.) or with a Myc-tagged RAD6A plasmid for 48 h and then incubated with 25 μM MG132 for another 10 h. Immunoprecipitation was then performed using an anti-ASF1A antibody under denaturing conditions. The results indicated that RAD6 overexpression indeed increased the ASF1A polyubiquitination levels (Figure 1F).

From these results, we conclude that RAD6 promotes the degradation of ASF1A through the ubiquitin-proteasome pathway in human cells.

### MDM2 is the corresponding E3 ligase for ASF1A degradation in homo sapiens

**Figure 2:**
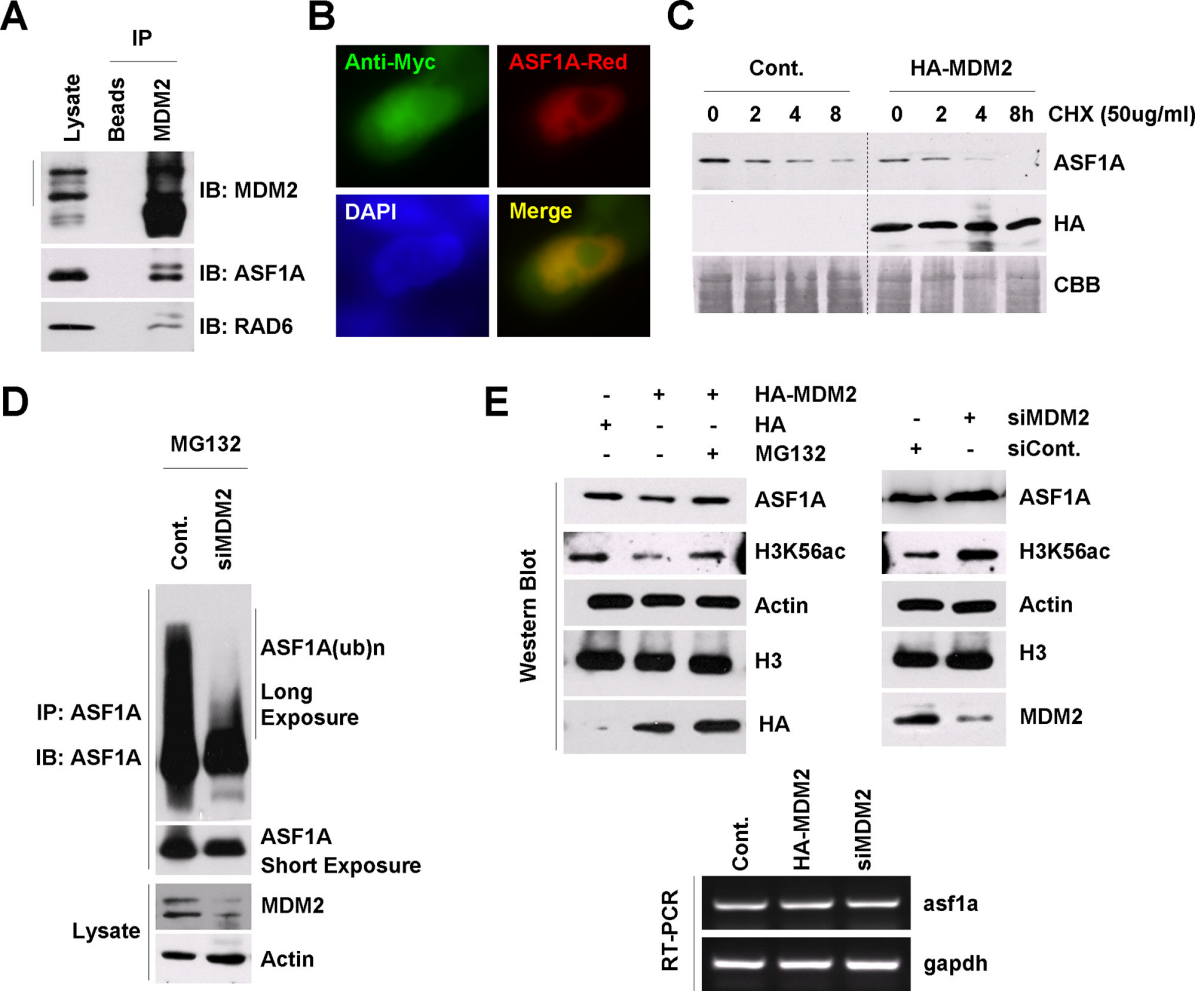
MDM2 regulates ASF1A ubiquitination and degradation in *Homo sapiens* **A.** MDM2 interacts with ASF1A and RAD6 *in vivo*. HL-7702 cells were lysed for an endogenous Co-IP assay with an anti-MDM2 antibody. Western blot assays were performed using anti-ASF1A or anti-RAD6 antibodies to detect the interaction between MDM2 and ASF1A or MDM2 and RAD6 *in vivo*. **B.** MDM2 colocalizes with ASF1A in HL-7702 cells. HL-7702 cells transfected with MDM2-Myc and ASF1A-Red were used for an immunofluorescence assay with an anti-myc antibody (green). **C.** MDM2 promotes ASF1A degradation. HL-7702 cells were transfected with or without HA-tagged MDM2 for 48 h. The cells were then incubated with 50 μg/mL CHX for the indicated times. Cell lysates were prepared and subjected to western blot assays with antibodies as indicated. **D.** MDM2 regulates ASF1A ubiquitination. HL-7702 cells were transfected with control or MDM2-specific siRNA for 48 h as indicated. The cells were then incubated with 25 μM MG132 for another 10 h. An immunoprecipitation experiment was performed using an anti-ASF1A antibody under denaturing conditions. An anti-ASF1A antibody was used in a western blot assay to detect the ubiquitinated form of ASF1A. **E.** MDM2 regulates ASF1A protein levels and H3K56Ac levels through the 26s proteasome. HL-7702 cells were transfected with an HA-tagged MDM2 plasmid or a control HA empty vector or with MDM2-specific siRNA or control siRNA for 48 h. The cells were then lysed for western blot assays with antibodies as indicated (upper). HL-7702 cells were transfected with HA-tagged MDM2 or MDM2-specific siRNA for 48 h. Total RNA was isolated and subjected to an RT-PCR assay with primers specific for *asf1a* and *gapdh* genes (lower).

Next, we determined the corresponding E3 ligase for ASF1A degradation. We and other groups have reported that RAD6 can cooperate with MDM2 or other E3 ligases to regulate substrate degradation [45–49]. We therefore tested whether MDM2 participates in the ASF1A degradation process. HL-7702 cells were lysed for an endogenous Co-IP assay using an anti-MDM2 antibody; then, the precipitated proteins were subjected to western blot assays with an anti-ASF1A or anti-RAD6 antibody as indicated. From the results, we found that MDM2 interacted with ASF1A and RAD6 *in vivo* (Figure 2A). This result indicated that MDM2 likely participates in the regulation of ASF1A degradation by cooperating with RAD6. To determine whether MDM2 colocalizes with ASF1A in cells, we performed an immunofluorescence assay. HL-7702 cells transfected with a MDM2-Myc plasmid and an ASF1A-Red plasmid for 48 h were harvested and stained with an anti-Myc antibody and DAPI as indicated in Figure 2B. Our results indicated that MDM2 indeed colocalized with ASF1A in HL-7702 cells (Figure 2B). More over, our quantification suggested that almost all the transfected cells show this kind of colocalization pattern (data not shown).

We next performed a chase assay to determine the effect of MDM2 on the half-life of ASF1A. HL-7702 cells were transfected with an empty vector (Cont.) or with a HA-tagged MDM2 for 48 h; then, the cells were incubated with 50 μg/mL CHX for the indicated times, as shown in Figure 2C. We found that MDM2 overexpression also decreased the ASF1A half-life time, indicating an accelerated ASF1A degradation. To determine whether MDM2 regulates ASF1A polyubiquitination, we performed an *in vivo* ubiquitination assay. The result indicated that knockdown of MDM2 expression decreased ASF1A polyubiquitination levels (Figure 2D), while MDM2 overexpression increased ASF1A polyubiquitination (data not shown).

We also determined whether MDM2 regulates the ASF1A protein levels and ASF1A-regulated H3K56Ac levels. HL-7702 cells transfected with the HA empty vector or the HA-tagged MDM2 plasmid or a control siRNA or MDM2-specific siRNA for 48 h were treated with or without MG132 as indicated in Figure 2E. The cell extracts were then prepared and used for western blot and RT-PCR assays. From the result, we observed that MDM2 overexpression downregulated the ASF1A protein levels and H3K56Ac levels, while knockdown of MDM2 expression had an opposite effect on ASF1A protein levels and H3K56Ac levels (Figure 2E, upper). There was no obvious change in ASF1A mRNA levels when the MDM2 expression levels were altered (Figure 2E, lower).

From these results, we identified the corresponding E3 ligase for ASF1A degradation and found that MDM2 plays a critical role in the regulation of ASF1 degradation through the ubiquitin-proteasome pathway.

### RAD6 forms a ternary complex with MDM2 and ASF1A

**Figure 3:**
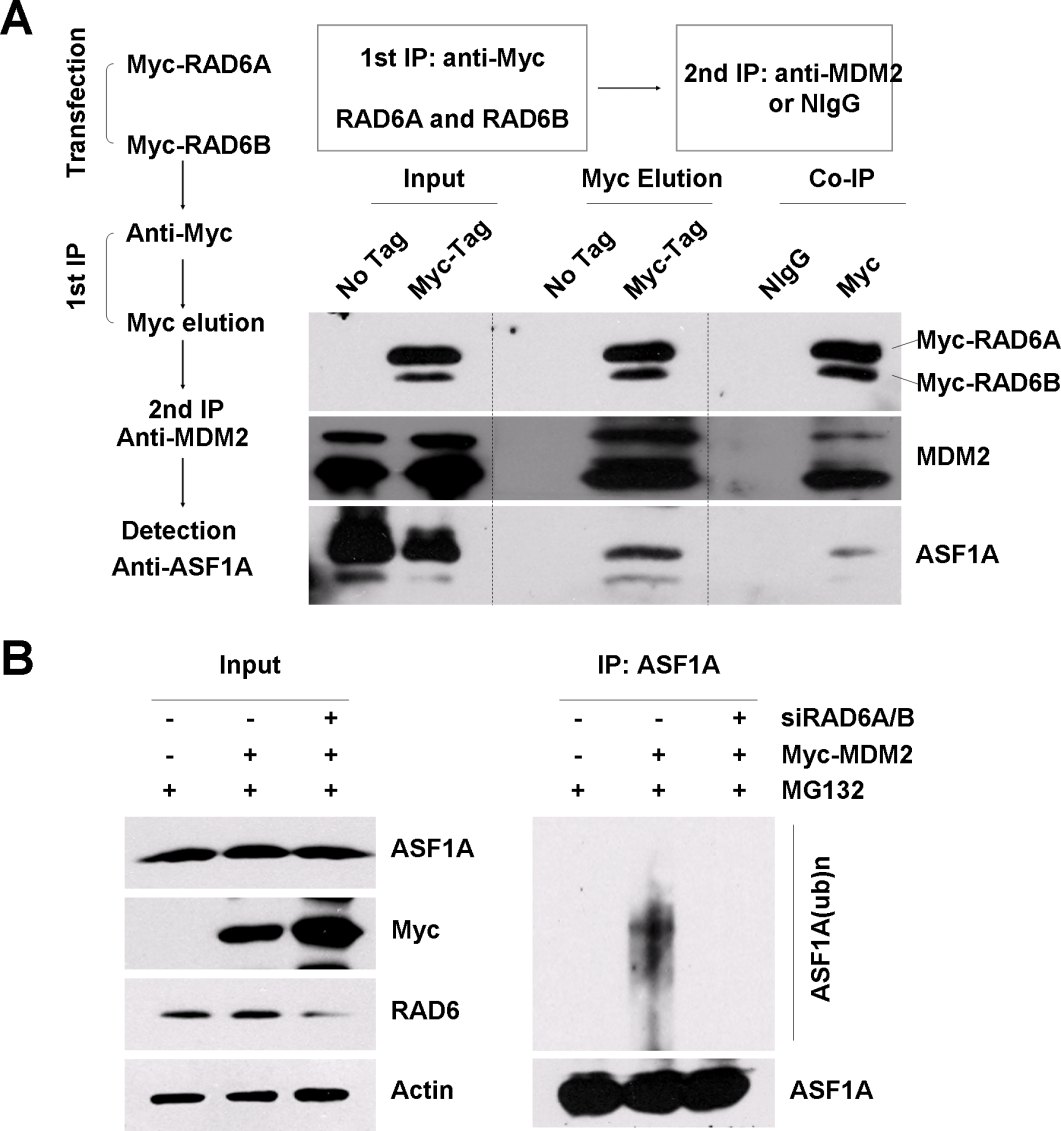
RAD6, MDM2 and ASF1A form a ternary complex **A.** A two-step co-immunoprecipitation experiment was performed to test for ternary complex formation. HL-7702 cells were transfected with or without (no tag) Myc-RAD6 (RAD6A and RAD6B). The first immunoprecipitation was performed with an anti-Myc antibody. The complex was eluted using the Myc peptide. The second immunoprecipitation step used an anti-MDM2 antibody or the control mouse IgG (NIgG) to precipitate the complex. The protein samples from each step were then separately subjected to western blot analysis using anti-Myc, anti-MDM2, and anti-ASF1A antibodies. **B.** MDM2-induced ASF1A polyubiquitination is dependent on the presence of RAD6 *in vivo*. HL-7702 cells were transfected with (+) or without (−) Myc-MDM2 together with (+) or without (−) of RAD6 siRNAs for 48 h and incubated with 25 μM MG132 for another 10 h. Immunoprecipitation analysis was performed under denaturing conditions with an anti-ASF1A antibody. The anti-ASF1A antibody was also used to visualize the amounts of precipitated ASF1A and ubiquitinated ASF1A in the following western blot assay. The expression levels of ASF1A, Myc-MDM2 (Myc) and RAD6 are shown on the left.

Both RAD6 and MDM2 regulate ASF1A degradation; we therefore investigated whether RAD6, MDM2 and ASF1A form a functional complex *in vivo*. To determine whether RAD6, MDM2, and ASF1A exist in the same complex, two-step co-immunoprecipitation experiments were performed (Figure 3A). HL-7702 cells were transfected with Myc-RAD6 plasmids; non-transfected cells were used as a negative control. In the first immunoprecipitation step, anti-Myc was used to pull down RAD6, and the Myc peptide (GenScript) was used to elute the complex. The eluate was then immunoprecipitated with an anti-MDM2 antibody or a control IgG followed by western blotting to detect ASF1A.

As shown in Figure 3A, ASF1A was present in the final immunoprecipitate but not in the control sample, confirming that RAD6, MDM2 and ASF1A existed in a ternary complex. We next examined whether the presence of RAD6 is essential for MDM2-induced ASF1A ubiquitination. HL-7702 cells were transfected with an empty vector (−) or without the Myc-MDM2 plasmid (+) in the presence (+) or absence (−) of RAD6 siRNAs (RAD6A and RAD6B) for 48 h; then, the cells were treated with 25 μM MG132 for another 10 h. The harvested cells were lysed and subjected to IP with an anti-ASF1A antibody under denaturing conditions. The IP lysates were then immunoblotted with an anti-ASF1A antibody. The results showed that RAD6 is required for MDM2 overexpression promoted ASF1A polyubiquitination (Figure 3B, compare lanes 4–6).

Taken together, our results show that RAD6, MDM2 and ASF1A form a functional complex regulating ASF1A polyubiquitination.

### RAD6-MDM2-regulated ASF1A degradation participates in tumorigenesis

It has been reported that ASF1A and H3K56Ac levels are elevated in many different cancer tissues [25]. We therefore questioned whether RAD6-MDM2-regulated ASF1A degradation participates in tumor development.

**Figure 4:**
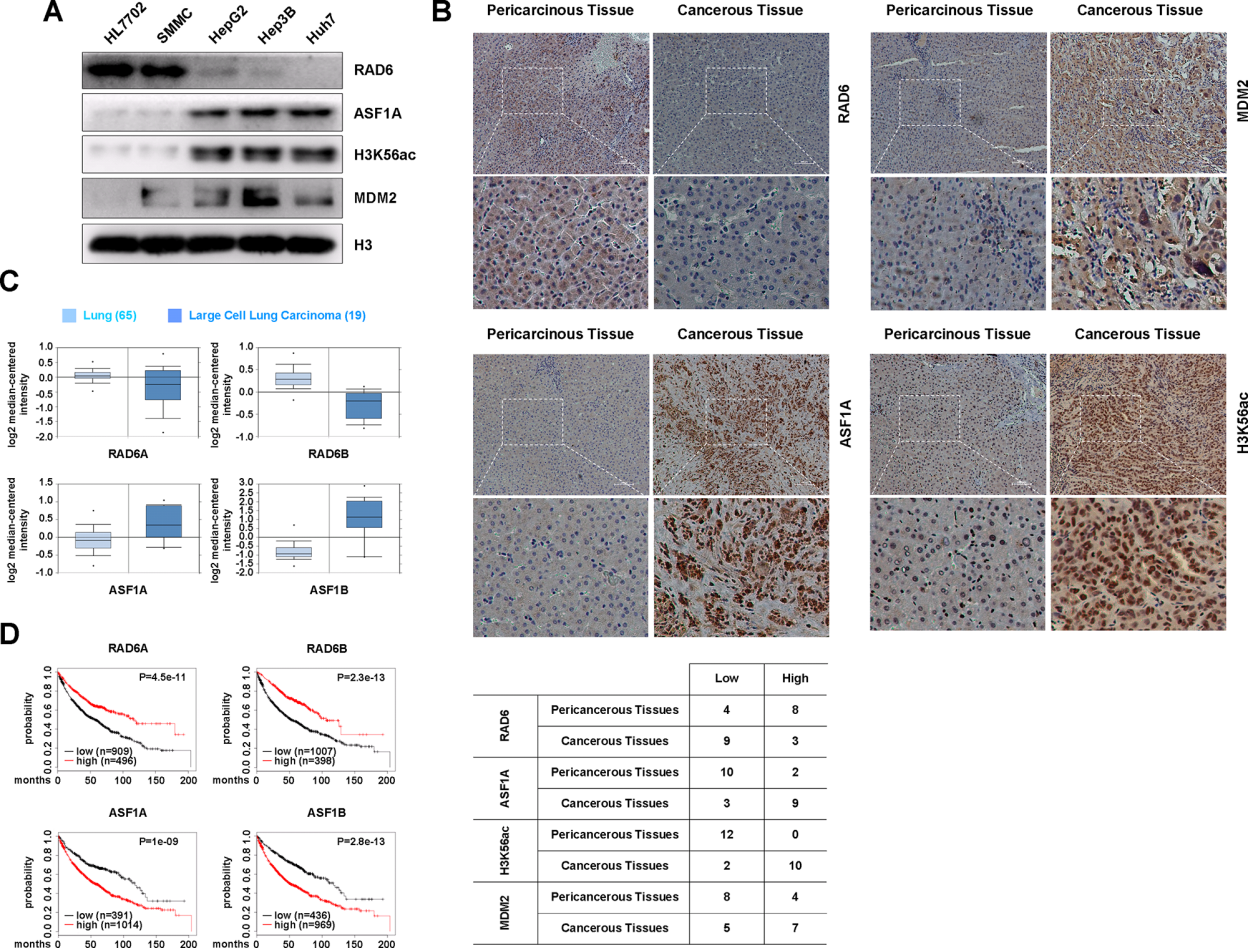
The regulation of ASF1A protein levels by the RAD6-MDM2 ubiquitin ligase participates in cancer development **A.** ASF1A protein levels and the related H3K56Ac levels are increased in hepatoma cell lines compared with normal liver cell line, while RAD6 protein levels are decreased in hepatoma cell lines. Different cell lines including normal liver cell line HL-7702 and four hepatoma cell lines were lysed and subjected to western blot analyses with antibodies as indicated. **B.** RAD6, ASF1A, H3K56Ac and MDM2 protein levels in human liver cancer samples. 12 pairs of human liver cancer samples were collected and subjected to immunohistology assay with specific antibodies as indicated. The quantification of the resultes were shown in the table below. **C.** Gene expressional assay was performed with the Oncomine online data base (http://www.oncomine.org). **D.** Bioinformatic analysis for the relevancies of RAD6 and ASF1 in lung cancer survival rate was performed using a clinic-based online Kaplan-Meier plot database (http://www.kmplot.com).

We first investigated the correlation between RAD6 and MDM2 protein levels and ASF1A and H3K56Ac levels in the normal liver cell line HL-7702 and in different hepatoma cell lines (SMMC, HepG2, Hep3B and Huh7). The results indicated that ASF1A protein levels and H3K56Ac levels were increased in hepatoma cell lines, while RAD6 protein levels were decreased in hepatoma cell lines (Figure 4A, upper). However, the E3 ligase MDM2 protein levels were also increased in hepatoma cells lines (Figure 4A, lower), which support its oncogenic role in tumorigenesis [51–53]. However, the decrease of RAD6 protein levels likely determined the upregulation of ASF1A and H3K56Ac levels although MDM2 levels were increased in hepatoma cell lines. This conclusion is consistent with our data shown in Figure 3B. The relationship between RAD6 and ASF1A protein levels showed a good reverse correlation and was consistent with the above results (RAD6 determines the polyubiquitination and degradation of ASF1A; Figure 1 and Figure 3B). Therefore, we propose that RAD6 may be the restricting factor in the determination of ASF1A ubiquitination and degradation.

To assess the clinical relevance between RAD6 and ASF1, we performed an immunohistology assay with 12 pairs of human liver cancer samples. We found that RAD6 protein levels decreased mostly in the cancer samples compared with the pericarcinous tissue, while the ASF1A, H3K56Ac and MDM2 levels increased significantly in most of the tumor samples (Figure 4B). This results are consistent to the data from cell lines (Figure 4A).

To further confirm the correlation between RAD6 and ASF1 expression levels in tumorigenesis, an Oncomine assay and a Kaplan-Meier plot survival assay were performed using clinic-based bioinformatic analyses. We found that both RAD6A and RAD6B showed downregulated expressional levels in human lung cancers compared with normal lung tissues, while the expression of both ASF1A and ASF1B are upregulated (Figure 4C). Furthermore, the Kaplan-Meier plot assay indicates that lower RAD6 expression levels and higher ASF1 expression levels significantly correlated with poor survival rate in lung cancer patients (Figure 4D). This result suggests that RAD6 and ASF1 are negatively correlated with regard to lung cancer patients. Besides, similar results were also obtained in ovarian cancer patients (data not shown) indicated that the negative correlation between RAD6 and ASF1A is possibly a general event occurred in tumorigenesis.

### RAD6 and ASF1A negatively correlated in cell proliferation and cell migration

**Figure 5:**
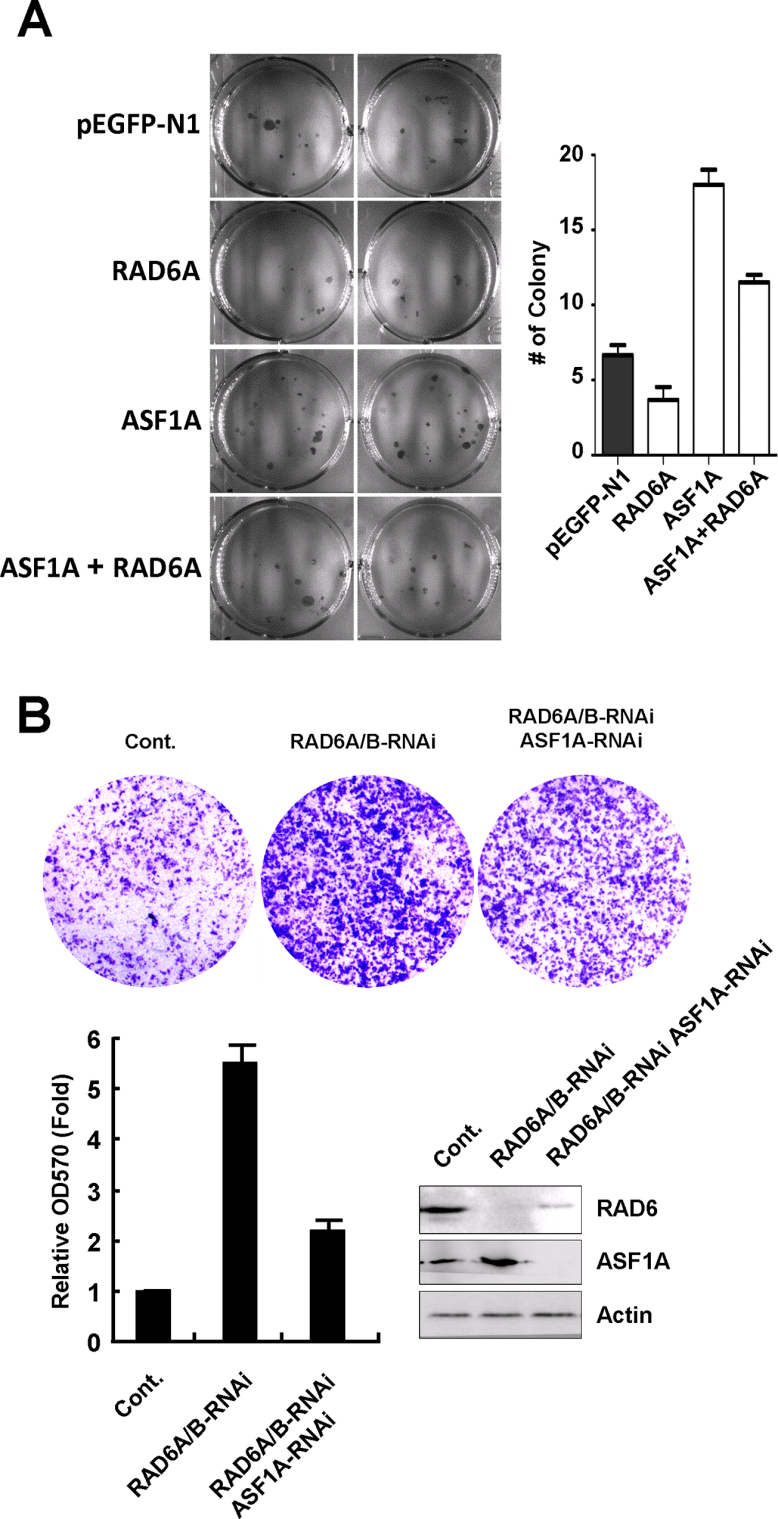
RAD6 and ASF1A negatively correlated in tumor cell proliferation and migration **A.** Human lung cancer H1299 cells were transfected with an GFP empty vector, or a RAD6A-GFP, or a ASF1A-Red, or a combination of these two plasmids, and stable cell lines were generated. The cells were then used for a soft agar assay to determine the ability of cell proliferation. The quantification of the soft agar data are shown on the right. **B.** Knockdown of RAD6 expression promotes liver cell migration in an ASF1A-dependent manner. HL-7702 cells were transfected with or without RAD6A and RAD6B-specific siRNAs together with or without an ASF1A-specific siRNA as indicated for 48 h. The cells were then used for a Transwell assay to determine the cell migration ability. The quantification of the Transwell data and related RNAi efficiency are shown below.

To shed light on the biological effect of the interplay between RAD6 and ASF1A in tumorigenesis, we next examined the effect of RAD6 and ASF1A on cell proliferation and cell migration. Human lung cancer cell line H1299 cells were transfected an GFP empty vector, or an GFP-tagged RAD6A plasmid, or a Red-tagged ASF1A plasmid, or RAD6A and ASF1A plasmids together. The stable cell lines were then used for soft agar assay. We found that overexpression of RAD6A inhibits the cell proliferation, while overexpression of ASF1A promotes the proliferation of H1299 cells (Figure 5A), suggesting an negative effect of RAD6 and ASF1A in tumor cell proliferation. Further interestingly, our results also showed that the combination of ASF1A overexpression with RAD6 overexpression efficiently rescued the effect of ASF1A overexpression on cell proliferation (Figure 5A), which is consistent to the degradative effect of RAD6 on ASF1A protein levels.

To analyze the effect of RAD6 and ASF1A on cell migration, HL-7702 cells were transfected with siRNAs specific for RAD6A and RAD6B or a control siRNA together with or without ASF1A-specific siRNA as indicated in Figure 5B for 48 h. The cells were then subjected to a transwell assay to determine their migration ability. The results showed that knockdown of RAD6 expression elevated the migration ability of HL-7702 cells significantly, while the double knockdown of RAD6 and ASF1A expression rescued the increased cell migration detected in RAD6 single-knockdown cells (Figure 5B).

### Drosophila dRad6 interacts with dASF1 both *in vitro* and *in vivo*

To understand whether the regulation of ASF1 by RAD6 is conserved, we next analyzed the relationship between dRad6 and dASF1 in drosophila system. We first determined the interaction between dRad6 and dASF1 both *in vitro* and *ex vivo*. As shown in Supplementary Figure S2A, a GST pull-down assay suggested that dASF1 was pulled down by the GST-dRad6 fusion protein. This result indicated that dRad6 interacts with dASF1 *in vitro*. To determine whether dRad6 interacts with dASF1 *ex vivo*, we performed co-immunoprecipitation (Co-IP) assays using *Drosophila* embryonic S2 cells transfected with HA-tagged dRad6 or GFP-tagged dASF1. Transfected S2 cells were then incubated with 50 μM MG132 for 10 h. The cells were lysed and subjected to Co-IP with antibodies against the HA or GFP tag. The precipitated proteins were then detected through western blot analysis using anti-dASF1 or dRad6 antibodies. The results showed that dRad6 forms a complex with dASF1 *ex vivo* (Supplementary Figure S2B).

As the interaction between dRad6 and dASF1 is very weak in normal drosophila S2 cells (only very high amount proteins can detect the interaction with Co-IP assay). We next investigated some stress stimulations can enhance the their interaction. DNA-damaging reagent MMS were used as RAD6 protein is recognized as a key node in the regulation of DNA damage repair. S2 cells transfected with GFP-dASF1 were treated with or without 0.1% MMS for 24 h. Then, the cells were lysed and subjected to a Co-IP assay with an anti-GFP antibody followed by immunoblotting with anti-dRad6 or anti-GFP antibodies as indicated. The results showed that MMS treatment significantly promoted the interaction between dRad6 and dASF1 (Supplementary Figure S2C, compare lanes 1 and 2). We further confirmed this result using immunofluorescence assays. S2 cells transfected with a DsRed2-tagged dRad6 plasmid together with a GFP-tagged dASF1 plasmid were treated with or without 0.1% MMS for 24 h; then, the cells were harvested and stained with DAPI (to detect the cell nuclei). The results showed that there was a substantial colocalization between dRad6 and dASF1 after MMS stimulation (Supplementary Figure S2D). We also analyzed the endogenous distribution of dRad6 and dASF1 with their specific antibodies, and similar results were obtained (Supplementary Figure S2D), supporting the enhanced interaction between dRad6 and dASF1.

### dRad6 regulates dASF1 degradation, especially after DNA damage

To address the function of this interaction, we first examined the effect of dRad6 or dASF1 depletion on dASF1 or dRad6 protein levels under normal conditions as indicated in Supplementary Figure S3A. S2 cells were incubated with or without dRad6 or dASF1 dsRNA for 6 days; then, cell extracts were prepared and subjected to western blot assays. The results showed no obvious effect on dASF1 or dRad6 protein levels under normal conditions after knockdown of dRad6 or dASF1 expression levels (Supplementary Figure S3A).

We next tested the changes in dASF1 mRNA levels after dRad6 depletion in S2 cells both in MMS-treated and untreated conditions. S2 cells incubated with an control dsRNA or with a dRad6 dsRNA for 6 days were treated with or without 0.1% MMS for 24 h. Total RNA was isolated and subjected to RT-PCR assays. The data showed no obvious effect of dRad6 depletion on dASF1 mRNA levels regardless of MMS treatment (Supplementary Figure S3B).

Next, we investigated whether dRad6 regulates dASF1 degradation. A chase experiment was employed to determine the effect of dRad6 on the dASF1 half-life (indicates the degradation rate) with or without MMS treatment. S2 cells incubated with a control or with a dRad6 dsRNA for 6 days were treated with or without 0.1% MMS for 24 h; then, the cells were used for a chase assay. As shown in Supplementary Figure S3C (upper four panels), dRad6 depletion had no significant effect on the dASF1 half-life (protein degradation) under normal conditions (without MMS treatment). However, MMS treatment promoted the degradation of dASF1, and this increased dASF1 degradation was in a dRad6-dependent manner. dRad6 depletion inhibited the increase of dASF1 degradation rate under 0.1% MMS treatment conditions (Supplementary Figure S3C, lower four panels). The quantification of the relative dASF1 protein levels is also shown in Supplementary Figure S3C (below). The result therefore supports a role of dRad6 in the regulation of dASF1 degradation after DNA damage.

We further tested the effect of dRad6 on dASF1 ubiquitination by an *in vivo* ubiquitination assay. S2 cells transfected with or without HA-tagged dRad6 were treated with or without 0.1% MMS for 14 h. Then, the cells were incubated with 50 μM MG132 for another 10 h. The cells were lysed and subjected to an IP assay with an anti-dASF1 antibody under denaturing conditions. As shown in Supplementary Figure S3D, dRad6 overexpression promoted dASF1 polyubiquitination significantly, especially after MMS treatment (compare lanes 1 and 2).

From the above results, we can conclude that the DNA damage reagent MMS promoted the interaction between dRad6 and dASF1 and further induced dASF1 degradation through the ubiquitin-proteasome pathway.

### dRad6 regulates H3K56Ac levels in response to DNA damage stimulation

We next questioned whether dRad6 affects H3K56Ac levels in normal and MMS-treated cells. Surprisingly, we found only a slight decrease in H3K56Ac when dASF1 was depleted in S2 cells (Supplementary Figure S4A). The depletion of dRad6 also causes no obvious change in H3K56Ac under normal conditions (Supplementary Figure S4B).

Since MMS stimulation promoted the interaction between dRad6 and dASF1 and the degradation of dASF1 (Supplementary Figure S2C and S2D, Supplementary Figure S3C and S3D), we further analyzed the effect of dASF1 depletion and dRad6 overexpression on H3K56Ac levels in response to MMS treatment. S2 cells were incubated with dASF1 dsRNA for 6 days and then treated with 0.1% MMS for the indicated times. The cells were lysed and subjected to western blot assays. The results showed that dASF1 depletion promoted H3K56Ac decrease significantly after MMS stimulation compared with the control group (Supplementary Figure S4C, “dASF1-RNAi” 3 lanes).

Next, we examined the effect of dRad6 on dASF1 and H3K56Ac levels in response to DNA damage. S2 cells transfected with HA-tagged dRad6 for 48 h were treated with 0.1% MMS for the indicated times. The cell extracts were prepared and subjected to western blot analyses. As shown in Supplementary Figure S4C (“dRAD6-HA” 3 lanes), dRad6 overexpression induced the downregulation of dASF1 protein levels after MMS treatment. The result is consistent with the conclusion in Supplementary Figure S3C and S3D (dRad6 promoted dASF1 ubiquitination and degradation after MMS treatment). In parallel to the change in dASF1, dRad6 overexpression also promoted H3K56Ac decrease in response to MMS treatment (Supplementary Figure S4C, “dRAD6-HA” 3 lanes). Furthermore, the MMS-induced decreases of dASF1 and H3K56Ac levels in dRad6-HA overexpressing S2 cells was abolished by MG132 treatment (Supplementary Figure S4C, “dRad6-HA+MG132” 3 lanes) supporting the observed effects of dRad6 on dASF1 and H3K56Ac levels are achieved through proteasome degradation pathway. The quantification of the corresponding band densities is also shown in Supplementary Figure S4C (lower diagram).

**Figure 6:**
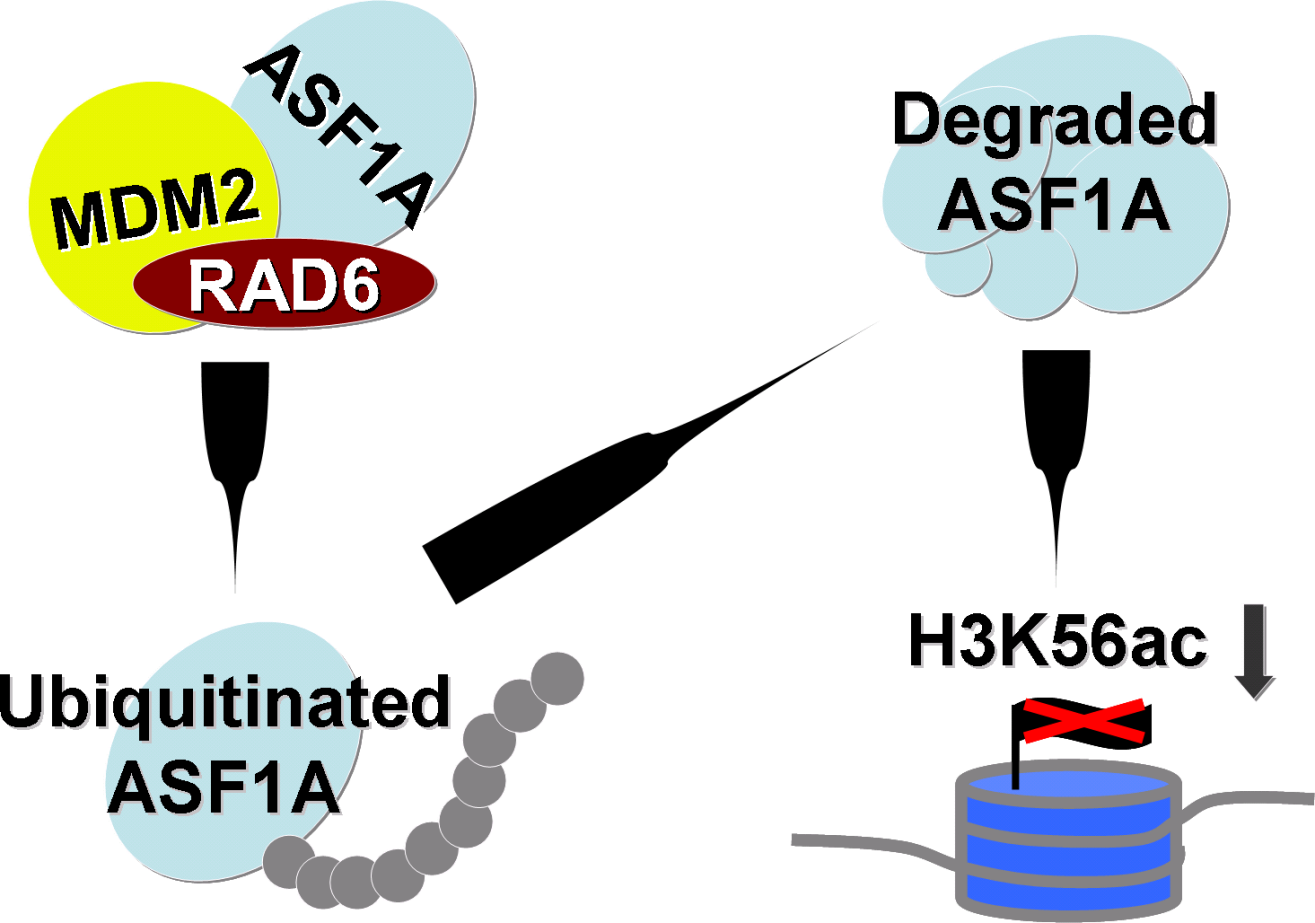
Working model RAD6 protein levels are decreased in cancer cells. The downregulation of RAD6 promotes ASF1A accumulation in cancer cells and further upregulates H3K56Ac levels.

Above all, we found that the E2 ubiquitin-conjugating enzyme, RAD6, is a new ASF1 interacting partner and regulator in both *Homo sapiens* and *Drosophila melanogaster*, suggesting a conserved role of these two proteins especially in tumorigenesis (Figure 6).

## DISCUSSION

ASF1 is a well-known histone H3/H4 chaperone that plays critical roles in the regulation of chromatin assembly and disassembly [9–13]. Increasing evidence indicate that ASF1 participates in a variety of biological processes such as transcriptional elongation, DNA synthesis and damage repair, and cell cycle progression [8, 14, 24, 25].

There are two ASF1 isoforms in mammals, ASF1A and ASF1B; however, the functions of these two proteins partially differ under physiological conditions. For example, ASF1B rather than ASF1A is highly expressed in the thymus and testis [54]. ASF1A, not ASF1B, regulates histone H3K56Ac levels and is increased in many tumor tissue types together with H3K56Ac levels [25]. In addition, it has been reported that ASF1B is necessary for breast cancer cell proliferation, indicating its prediction for the outcome of breast cancer patients [24]. Collectively, increasing data indicate that ASF1 significantly participates in the regulation of tumorigenesis and development. Therefore, the control of ASF1 to a proper protein level is important for its biological functions. However, the molecular mechanism of ASF1 degradation is poorly understood, especially in mammals [35].

In this work, we report for the first time that the RAD6-MDM2 ubiquitin ligase machinery regulates ASF1A degradation in human cells and that this regulation is evolutionarily conserved. From our data (Figure 1E and Figure 2C), we found that human ASF1A is a quick-turnover protein, with a half-life of less than 4 h. Detailed investigation showed that the RAD6-MDM2 ubiquitination machine regulated the polyubiquitination and further degradation of ASF1A. This finding was supported by the following evidence: 1) the E2 ubiquitin-conjugating enzyme RAD6 formed a complex with ASF1A and affected ASF1A polyubiquitination and degradation; 2) the E3 ligase MDM2 also formed a complex with ASF1A and affected ASF1A polyubiquitination and degradation; and 3) RAD6, MDM2 and ASF1A formed a functional ternary complex that allowed for ASF1A ubiquitination.

From our results (Figure 3B and Figure 4A), we also found that RAD6 was the restricting factor in the ASF1A ubiquitination and degradation process. ASF1A accumulated in cancer cell lines with downregulated RAD6 levels, although the MDM2 levels were upregulated (Figure 4A). This result is consistent with the result shown in Figure 3B. When RAD6 expression was knocked down, the ASF1A polyubiquitination induced by MDM2 overexpression was inhibited efficiently. *Drosophila* dASF1 has a substantially longer half-life (more than 4 h) compared with human ASF1A under normal conditions. This is likely because of the different cellular distribution pattern between *Drosophila* dRad6 and *Homo* RAD6. dRad6 was mainly localized in the cytoplasm without DNA damage stimulation (Supplementary Figure S2D), while human RAD6 was mainly localized in the nucleus (Figure 1B). When *Drosophila* S2 cells encounter DNA damage (such as MMS treatment), a dramatic nuclear translocation of dRad6 occurred (Supplementary Figure S2D), which promoted the interaction between dRad6 and dASF1 and dASF1 degradation. Therefore, a reduced half-life was found in MMS-treated S2 cells, and this increased degradation rate occurred in a dRad6-dependent manner (Supplementary Figure S3C).

It has been reported that H3K56Ac levels are regulated by ASF1 in yeast, *Drosophila* and humans (ASF1A, not ASF1B) [25, 26, 28]. We examined the relationships among RAD6/MDM2, ASF1 and H3K56Ac both in *Drosophila* and *Homo sapiens*. We noticed a very good correlation among these three factors. When we overexpressed RAD6, ASF1A protein levels decreased together with H3K56Ac levels (Figure 1C). MDM2 overexpression also induced a decrease in ASF1A protein levels and H3K56Ac levels in a proteasome-dependent manner, while the knockdown of MDM2 expression had an opposite effect (Figure 2E). However, in *Drosophila*, our results indicated that H3K56Ac levels were only partially dependent on the presence of dASF1 under normal conditions (Supplementary Figure S4A). Our study suggested that the presence of dASF1 was necessary for the maintenance of H3K56Ac levels under DNA damage conditions (Supplementary Figure S4C). dRad6 overexpression promoted a decrease in dASF1 protein levels and H3K56Ac levels after DNA damage in a proteasome-dependent manner. This result is consistent with the regulation of dASF1 protein levels by dRad6 (Supplementary Figure S3C and S3D).

Together, our study reveals that dASF1/ASF1A protein levels are regulated by the conserved dRad6/RAD6-MDM2 ubiquitin ligase machinery through the ubiquitin-proteasome pathway in *Drosophila* and *Homo sapiens*.

## MATERIALS AND METHODS

### Cell culture and transfection

The normal liver cell line HL-7702 and the hepatoma cell lines SMMC, HepG2, Hep3B, and Huh7 were cultured at 37°C in DMEM (Gibco) supplemented with 10% fetal bovine serum and antibiotics (penicillin and streptomycin) in a 5% CO_2_ incubator. The transfection of constructs into HL-7702 cells was performed with Lipofectamine 2000 (Invitrogen), according to the manufacturer's standard protocol.

### Plasmid constructs

The pIB-HA (Invitrogen) or the pET(42)b-GST (Clontech) plasmids expressing dRad6 and the pIB-GFP or pIB-His (Invitrogen) plasmids expressing dASF1 were constructed by cloning dRad6 and dASF1 cDNA into the pIB-HA or pET(42)b-GST vector and the pIB-GFP or pIB-His vector, respectively. The pCMV-Myc and pEGFP-N1 (Clontech) plasmids expressing RAD6A and RAD6B were constructed by cloning the RAD6A and RAD6B PCR products, which were amplified from HL7702 cell cDNA, into the pCMV-Myc and pEGFP-N1 vectors. The pDsRed2-N1 (Clontech) plasmid expressing ASF1A was constructed by cloning ASF1A cDNA amplified from HL7702 cells into the pDsRed2-N1 vector. The HA-MDM2 plasmid was a kind gift from Dr. Zhenkun Lou.

### RNAi knockdown of RAD6A, RAD6B and MDM2 in HL-7702 cells

siRNAs against RAD6A and MDM2 were designed and synthesized by GenePharm. RAD6B siRNA was purchased from Santa Cruz Biotechnology (Lot# SC-106915). siRNA against ASF1A was designed according to Groth et al., 2005 and 2007 [14, 18]. The RNAi efficiency was analyzed using RT-PCR.

RNAi transfection into HL-7702 cells was performed according to the manufacturer's protocol (Invitrogen). Briefly, 5 μg of each siRNA was transfected with 8 μl Lipofectamine 2000 (Invitrogen) per well of a 6-well plate.

### Co-immunoprecipitation analysis

S2 cells were transfected with pIB-dRad6-HA or pIB-dASF1-GFP, and HL-7702 cells were transfected with Myc-tagged RAD6A or RAD6B or Myc-tagged MDM2 using Lipofectamine 2000 (Invitrogen). After 48 h, the cells were harvested, washed with ice-cold PBS, resuspended in ATM lysis buffer (containing 100 mM Tris-Cl, pH 7.5, 150 mM NaCl, 0.2 mM EDTA, 20% glycerol, 0.4% NP-40, 2% Tween-20 and 0.2 mM PMSF) and sonicated on ice 10 times (3 s each), with 20% efficiency. The cell lysates were incubated with normal mouse IgG (Santa Cruz Biotechnology, as a negative control) or anti-HA (Zhongshan Golden Bridge), anti-Myc (Zhongshan Golden Bridge), or anti-MDM2 (Santa Cruz Biotechnology) antibodies at 4°C overnight. Protein A/G agarose beads were then added, and the solution was incubated for another 3 h, followed by centrifugation to harvest the agarose beads after they were washed 5 times with lysis buffer. The precipitated proteins were released by boiling in loading buffer and resolved by SDS-PAGE (15%). Immunoblot analyses were performed with antibodies against the Myc-tag, ubiquitin, p53 or MDM2.

### Two-step co-immunoprecipitation

Two-step co-immunoprecipitation was performed essentially according to the procedures described by Rui Y et al., 2004 [55]. Briefly, HL-7702 cells were transfected with Myc-RAD6A and Myc-RAD6B. Non-transfected HL-7702 cells were used as a negative control for the first immunoprecipitation. At 48 h after transfection, the cells were lysed with ATM lysis buffer, sonicated briefly, and centrifuged. The supernatant was incubated with an anti-Myc antibody bound to protein A/G-agarose beads for 4 h at 4°C. The beads were washed with lysis buffer three times, and the Myc-RAD6 protein complex was eluted with 300 μl of lysis buffer containing 250 mM NaCl and 250 μg/mL Myc peptide for 3 h at 4°C. The second immunoprecipitation was performed using 150 μl of eluate from the first immunoprecipitation with 350 μl of lysis buffer containing 464 mM NaCl and 2 μg of an anti-MDM2 antibody or the control IgG, followed by the addition of protein A/G-agarose beads.

### Western blot analyses

HL-7702 cells were lysed in ATM lysis buffer (containing 100 mM Tris-Cl, pH 7.5, 150 mM NaCl, 0.2 mM EDTA, 20% glycerol, 0.4% NP-40, 2% Tween-20 and 0.2 mM PMSF). The protein concentration of the supernatant was measured with a BCA Assay Kit (Calbiochem). Then, SDS-PAGE was performed using a 15% gel to resolve the proteins. Different amounts of total protein were loaded in each experiment to facilitate the detection of different target proteins. After electrophoresis, the proteins were transferred onto PVDF membranes (Amersham) and hybridized with primary antibodies at a dilution of 1:2,000. The HRP-labeled secondary antibodies (Zhongshan Golden Bridge) were used at a dilution of 1:4,000. An ECL detection system (Amersham) was used to detect the signals on the membranes.

### Immunofluorescence staining

Immunofluorescence staining was performed according to Ni JQ et al., 2006 [56]. The primary antibody used in this manuscript was rabbit anti-dRad6 (1:50), mouse anti-His (Zhongshan Golden Bridge, 1:50), and rabbit anti-MDM2 (Santa Cruz Biotechnology; 1:50). DAPI (Sigma) was used at a concentration of 1 × 10 ^−4^ μg/μL. The secondary antibody coupled to Texas red and FITC was purchased from the Zhongshan Golden Bridge Company, China (1:100). Images were captured using a laser scanning confocal microscope (Leica) with a 100× oil-immersion objective.

### RT-PCR assay

A total of 4 × 10^6^ HL-7702 cells were lysed to isolate total RNA using TRIzol reagent (Invitrogen), according to the manufacturer's instructions. Reverse transcription was performed as described by Ni JQ et al., 2006 [56]. Total RNA (5 μg) was reverse transcribed to synthesize cDNA in a volume of 20 μL (Reverse Transcriptase M-MLV, Takara). For each 25 μL PCR reaction, 1 μL of cDNA was used for 20–25 cycles. The PCR products were loaded onto a 2% agarose gel, stained with ethidium bromide and imaged.

### Cell proliferation assay (soft agar)

For the soft agar assay, the human lung cancer H1299 stable cells lines (transfected with GFP empty vector or RAD6A-GFP, or ASF1A-Red, or RAD6A and ASF1A together) were suspended in DMEM that contained 0.35% low melting agarose. The cells were then plated onto solidified 0.6% agarose in DMEM in 6-well culture plates at a density of 500 cells/well.

### Cell migration assay (Transwell)

Cell migration assay was performed with Transwell migration chambers (24-well, 8-μm pore size; Corning, Inc.) according to the manufacturer's instructions. The inner surface of the membrane was precoated with 40 μg/ml type I collagen. DMEM with 15% serum was used in the lower chamber to induce cell migration. Approximately 3 × 10^4^ cells in DMEM containing 0.5% serum were added into the upper chamber. After incubation at 37°C for 24 h, the non-migrating cells were wiped from the upper surface of the filter with a cotton swab. The cells that had migrated to the lower surface of the filter were stained with crystal violet and imaged.

## SUPPLEMENTARY MATERIALS AND METHODS FIGURES


