# Supplementary material for: A conserved RAD6-MDM2 ubiquitin ligase machinery targets histone chaperone ASF1A in tumorigenesis
Source: Oncotarget. 2015 Aug 22;6(30):29599–613. doi: 10.18632/oncotarget.5011 (PMC4745749; doi:10.18632/oncotarget.5011)
Supplement: Supplementary file 1 [file oncotarget-06-29599-s001.pdf]

SUPPLEMENTARY MATERIALS AND METHODS

RNAi knockdown in S2 Cells

The coding sequences of Drosophila dRad6 and dASF1 were first amplified with primers containing the gene sequence plus the sequence of a T7 promoter at the 5′-terminal end. dsRNA2 was prepared using a MEGAscript T7 kit (Ambion) according to the manufacturer's standard protocol. The products were quantified and stored at –80°C. RNAi in Drosophila S2 cells was performed according to the protocol of the Dixon laboratory and Ni JQ et al., 2006 [1].

REFERENCE

1. Ni JQ, Liu LP, Hess D, Rietdorf J, Sun FL. Drosophila ribosomal proteins are associated with linker histone H1 and suppress gene transcription. Genes Dev. 2006; 20:1959–1973.

SUPPLEMENTARY FIGURES

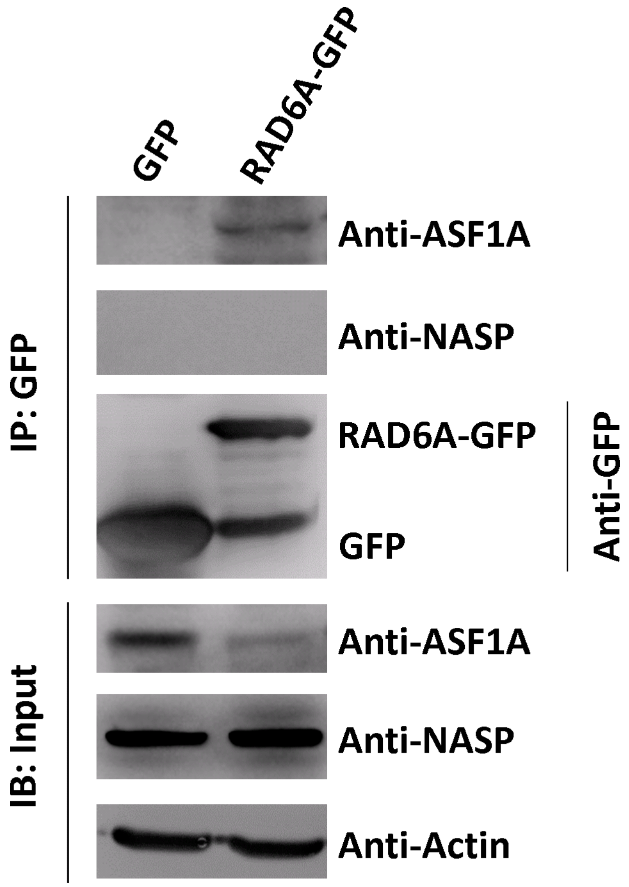

Supplementary Figure S1: RAD6 does not interact with histone chaperon NASP *in vivo*. HEK293T cells were transfected with an GFP empty vector or a RAD6-GFP plasmid as indicated for 48 h. Cells extracts were then prepared and subjected to Co-IP assay with anti-GFP antibody. The precipitants were further used for western blot assay with anti-NASP antibody or anti-ASF1A antibody.

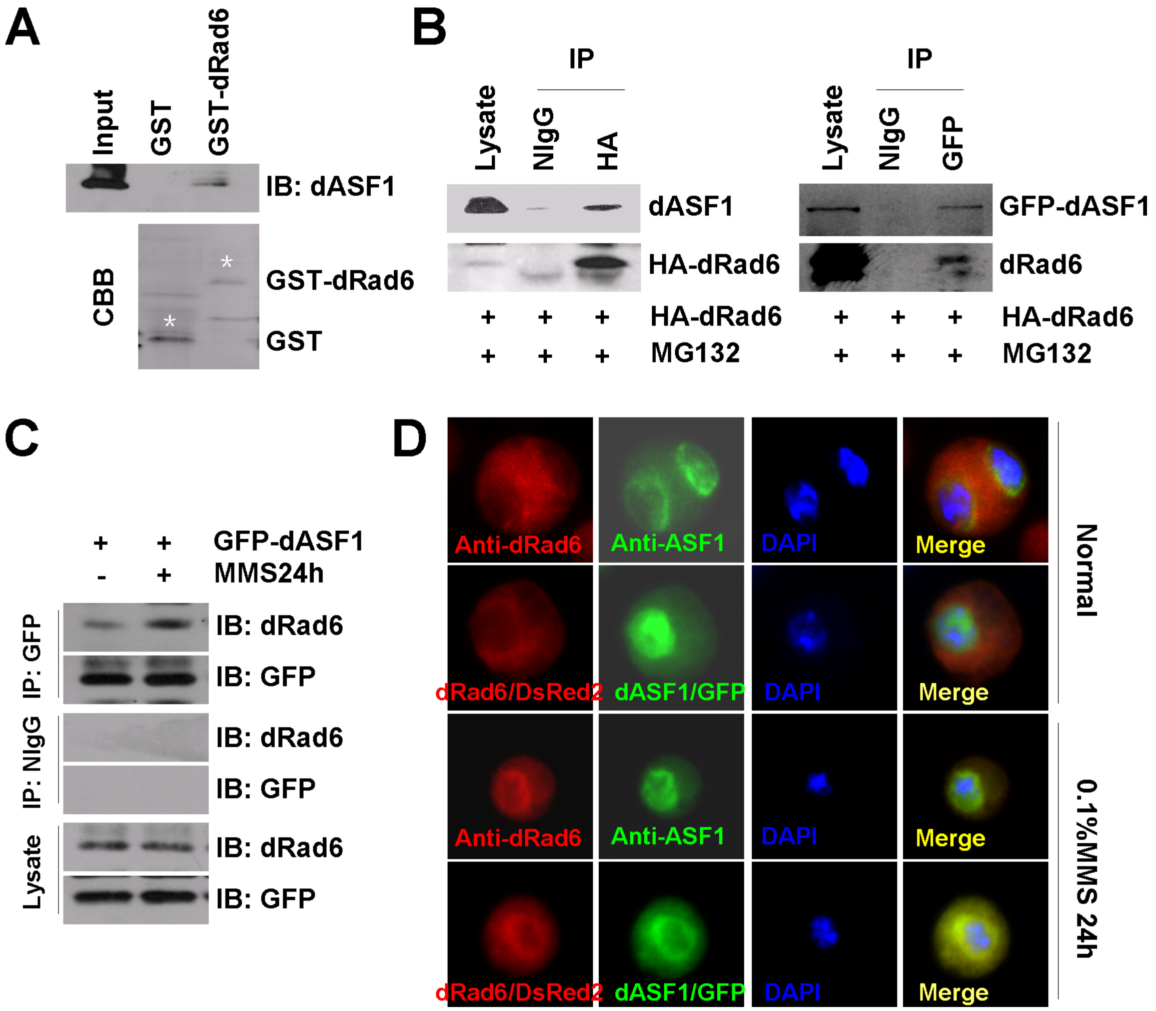

Supplementary Figure S2: ***Drosophila*** **dRad6 interacts with dASF1** ***in vitro*** **and** ***in vivo*****. A.** dRad6 interacts with dASF1 *in vitro*. A GST pull-down assay was performed with purified GST-dRad6, and the dASF1 antibody was used to detect their interaction. **B.** dRad6 interacts with dASF1 *in vivo*. S2 cells were transfected with HA-dRad6 for 48 h, and the cells were then incubated with 50 µM MG132 for another 10 h. The cells were lysed, and co-immunoprecipitation analysis was performed with an anti-HA antibody. An anti-dASF1 antibody was used to determine their interaction (left). For the reverse co-immunoprecipitation experiment, S2 cells were transfected with GFP-dASF1 for 48 h, and the cells were then incubated with 50 µM MG132 for another 10 h. The cells were lysed, and co-immunoprecipitation analysis was performed with an anti-GFP antibody. An anti-dRad6 antibody was used to determine their interaction (right). **C.** MMS treatment promotes the interaction between dRad6 and dASF1 *in vivo*. S2 cells transfected with GFP-dASF1 for 12 h were rinsed and stimulated with 0.1% MMS for 24 h with fresh medium. Then, the cells were lysed and subjected to a co-immunoprecipitation assay with an anti-GFP antibody, and an anti-dRad6 antibody was used to detect the interaction. **D.** Immunofluorescence analysis was performed to detect the localization of dRad6 and dASF1 in S2 cells. For exogenous immunofluorescence, S2 cells were cotransfected with DsRed2-dRad6 and GFP-dASF1 plasmids, and DAPI staining was used to visualize the cell nuclei. For endogenous assay, S2 cells were stained with anti-dRad6 antibody (Red) and anti-dASF1 antibody (green) as indicated.

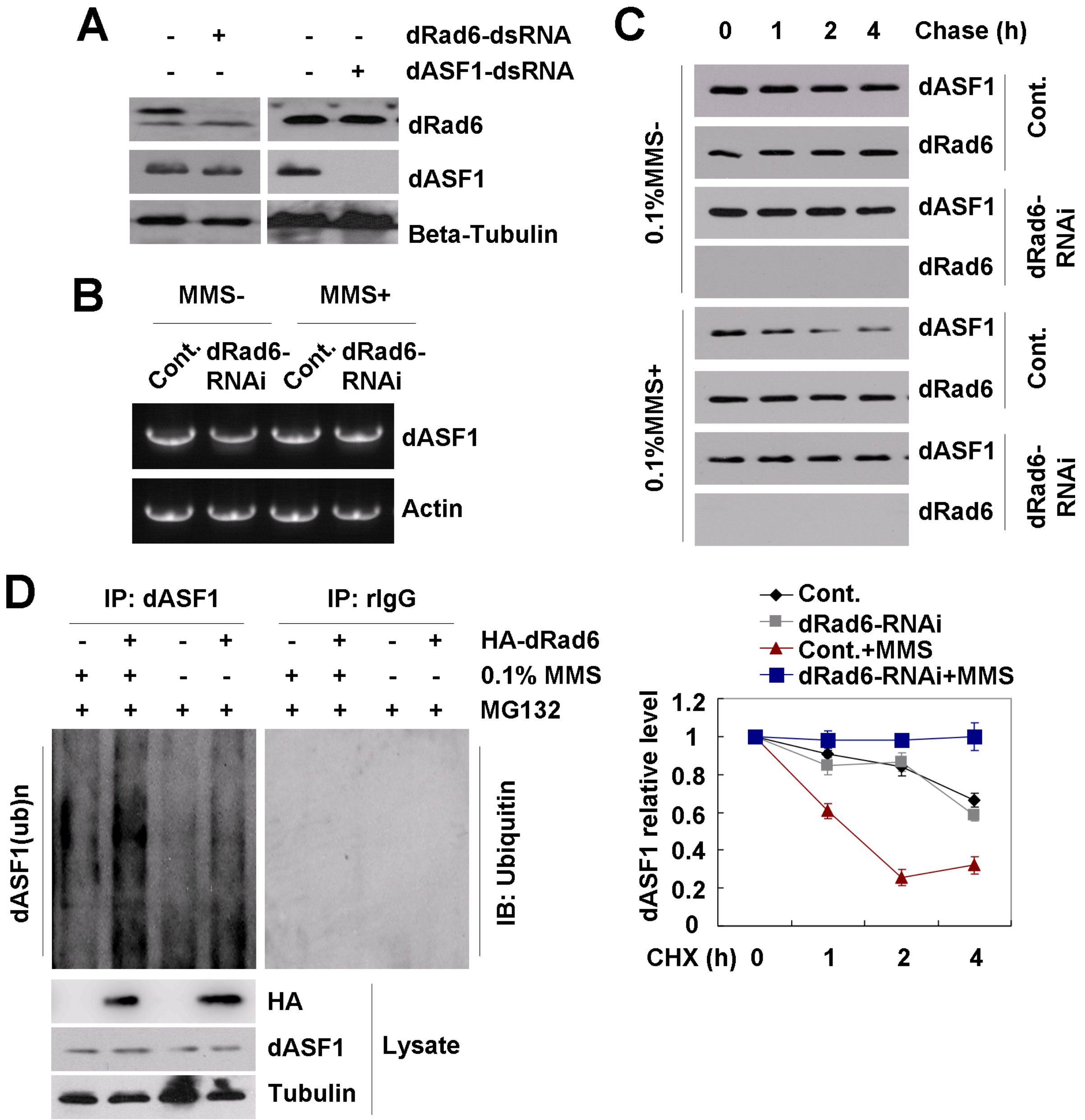

Supplementary Figure S3: dRad6 promotes dASF1 degradation and ubiquitination under DNA damage status. A. S2 cells were treated with dRad6 or dASF1 dsRNA for 6 days to knockdown the expression of the specific genes. Cell extracts were prepared, and anti-dASF1 and anti-dRad6 antibodies were used to detect changes in the corresponding proteins. **B.** RT-PCR analysis was performed to determine the effect of dRad6 depletion on dASF1 mRNA levels both in MMS treated or untreated cells. S2 cells were incubated with dRad6 dsRNA for 6 days. The cells were treated with or without 0.1% MMS for 24 h. Total RNA was then isolated and subjected to RT-PCR. **C.** dRad6 depletion inhibits dASF1 degradation after MMS stimulation. S2 cells incubated with dRad6 dsRNA for 6 days were treated with or without 0.1% MMS for 24 h. The cells were then incubated with 50 µg/mL CHX for the indicated times. The cell extracts were prepared and subjected to western blot assays with antibodies as indicated. The quantification of the corresponding band densities is shown below. **D.** dRad6 promotes dASF1 ubiquitination, especially after MMS stimulation. S2 cells transfected with HA-tagged dRad6 were treated with or without 0.1% MMS for 14 h. The cells were then incubated with 50 µM MG132 for another 10 h. An immunoprecipitation assay was performed with an anti-dASF1 antibody under denaturing conditions. The precipitated proteins were subjected to western blot analysis with an anti-ubiquitin antibody to detect the ubiquitinated form of dASF1.

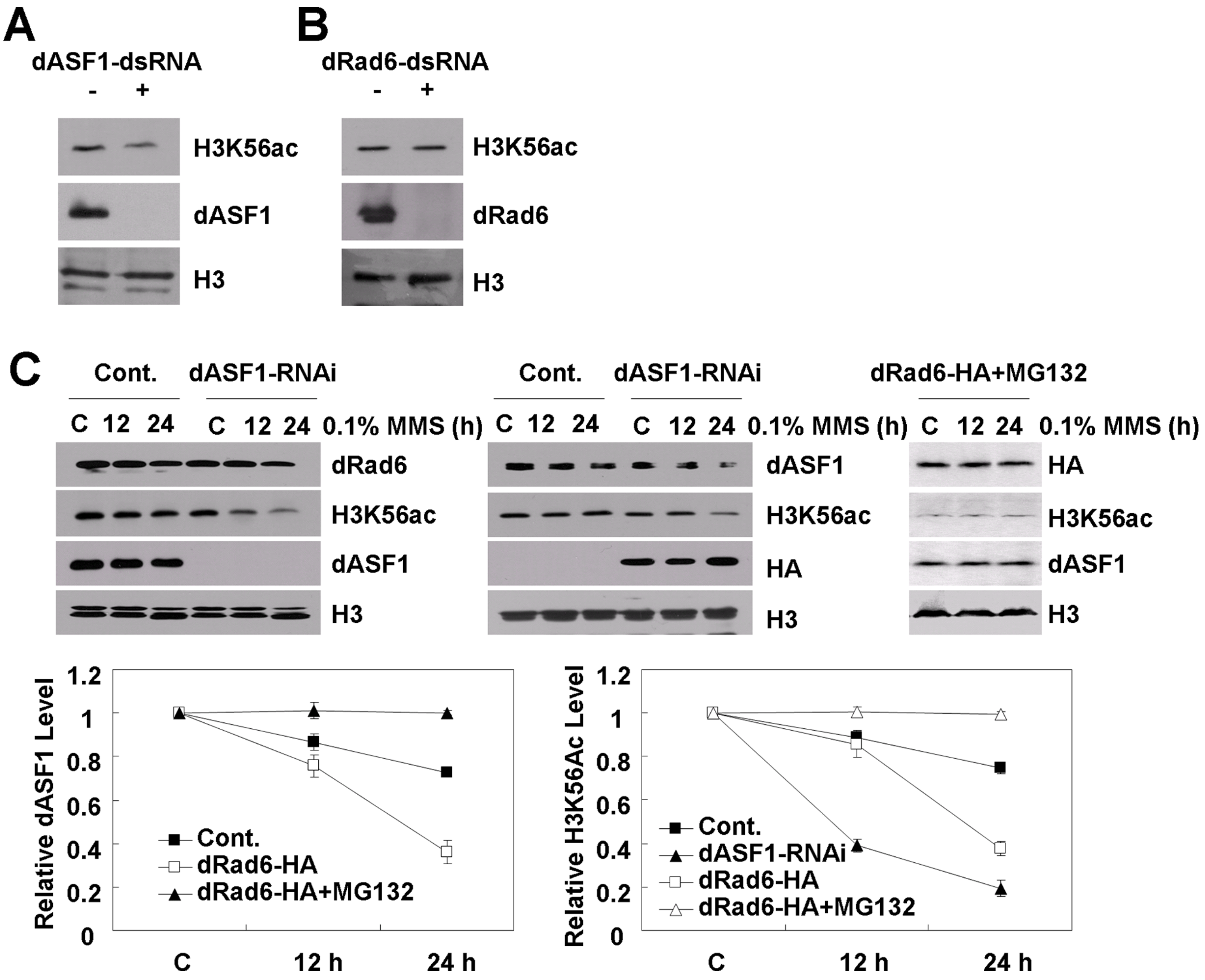

Supplementary Figure S4: dRad6 regulates H3K56Ac levels after DNA damage. A. S2 cells were treated with dASF1-specific dsRNA for 6 days to knock down the expression of dASF1. Cell extracts were prepared and subjected to western blot assays with antibodies as indicated. **B.** S2 cells were treated with dRad6-specific dsRNA for 6 days to knock down the expression of dRad6. Cell extracts were prepared and subjected to western blot assays with the antibodies as indicated. **C.** The effects of dASF1 depletion and dRad6 overexpression on H3K56Ac levels under normal or MMS stimulated conditions. S2 cells were incubated with dASF1 dsRNA for 6 days or transfected with HA-tagged dRad6 for 48 h. Next, the cells were treated with or without 0.1% MMS for the indicated times. The cell extracts were prepared and subjected to western blot assays with antibodies as indicated. The quantification of the corresponding band densities is shown below.
